# Chemical Assessment and Antimicrobial and Antioxidant Activities of Endophytic Fungi Extracts Isolated from *Costus spiralis* (Jacq.) Roscoe (Costaceae)

**DOI:** 10.1155/2014/190543

**Published:** 2014-12-17

**Authors:** Poliana Guerino Marson Ascêncio, Sérgio Donizeti Ascêncio, Aline Aires Aguiar, Adriana Fiorini, Raphael Sanzio Pimenta

**Affiliations:** ^1^Research Laboratory of Natural Products, Federal University of Tocantins, Avenida NS 15, 109 Norte, 77020-210 Palmas, TO, Brazil; ^2^Department of Clinical Analysis and Biomedicine, State University of Maringá, Avenida Colombo 5790, 87020-900 Maringá, PR, Brazil; ^3^Laboratory of General and Applied Microbiology, Federal University of Tocantins, Avenida NS 15, 109 Norte, 77020-210 Palmas, TO, Brazil

## Abstract

*Costus spiralis* (Costaceae) is a species native to the Amazon region and is used in traditional medicine. The endophytic fungi used in this study were obtained from leaves of this plant. 13 strains were selected to obtain hydroethanolic extracts and were submitted to hydroalcoholic extraction and evaluated for antioxidant activity by DPPH (2,2-difenil-1-picrilhidrazil) and FRAP (ferric reducing antioxidant power), and all of the fungi had positive results. The antimicrobial action of crude extracts had a good range of activities. All extracts had inhibitory activities against the yeasts of *Candida albicans* and *C. parapsilosis*, with 125 to 500 *μ*g/mL MIC. Eight extracts had antimicrobial activities against *Bacillus subtilis* (MIC from 62.4 to 125 *μ*g/mL), 5 against *Pseudomonas aeruginosa* (MIC from 125 to 500 *μ*g/mL), 2 against *Salmonella enterica* (MIC from 125 to 62.5 *μ*g/mL), and 2 against *Enterococcus faecalis* (MIC from 500 to 125 *μ*g/mL). The presence of secondary metabolites, including coumarins, was observed during chemical evaluation by thin layer chromatography. Total phenol content was estimated, and a strong positive correlation to antioxidant activity was observed, according to its Pearson coefficient. This is the first report of the bioactive potential of endophytic fungi isolated from the Costaceae family in Brazilian ecosystems.

## 1. Introduction

*Costus spiralis* is a Brazilian Amazon plant that is recognized for its medical and ornamental values. This plant is commonly used in popular medicine to treat urinary infections and kidney stones and for diabetes management. The plant also acts as an antioxidant, antibacterial agent and diuretic and to promote wound healing [1–3]. Normally, plants from tropical regions have a higher diversity of endophytic microorganisms compared with those found in temperate regions [4]. Endophytic fungi are microorganisms that colonize the vegetal tissues, either inter- or intracellularly, without causing any damage to the vegetal host. There are few studies that focus on the diversity, ecology, and other factors involved in endophyte plant interaction [5–7]. These microorganisms are a good resource for identifying new bioactive products, with more than 20,000 substances described to date [8]. Among these substances, 51% had novel structures and 80% exhibited some biological activity making endophytes a promising resource for the identification of new bioactive molecules [9, 10]. A variety of pharmacological activities have already been described from endophytic fungi, including antibacterial [11–13], antifungal [14–16], antiparasitic [17], trypanocidal [18, 19], leishmanicidal [19], antimalarial [20], anti-inflammatory [21], neuroprotective [22], antioxidant [7], immunosuppression [23], antiviral [23, 24], anticolinesterasic [12], antineoplastic [25–29], and cytotoxic [12, 22, 30] properties.

Despite scientific advances, infectious diseases remain a major contributor to mortality and morbidity in public health. The main explanation for this is the ability of microorganisms to acquire resistance against antimicrobial substances. This characteristic results in a constant need to discover and develop new drugs [31].

Oxygen radicals and superoxide anions play important roles in biochemical/physiological reactions in the human body. However, when these species are produced in excess due to pathophysiological processes or environmental interference, they can promote tissue damage and result in disease [32]. Currently, few antioxidant substances can be used in clinical situations, thus underscoring the necessity to identify new, safe, and efficient molecules for this purpose [33]. Bioactive molecules from new sources, such as endophytic fungi, deserve attention because they may lead to drugs with different pathologies, food additives, or cosmetics.

## 2. Materials and Methods

### 2.1. Endophytic Fungi

Endophytic fungi were isolated from healthy leaves of* C. spiralis*. From each leaf three 0.5 cm^2^ pieces were cut. The leaf sample fragments were surface sterilized by successive dipping in 2% Extran detergent (Sigma-Aldrich) for 2 min, 70% ethanol for 1 min, and 2% sodium hypochlorite for 3 min and then by washing with sterile distilled water for 2 min; they were then placed on potato dextrose agar (PDA; Difco) containing 100 mg/mL chloramphenicol. The sterile water wash was plated on PDA to confirm external disinfection. Plates were incubated for up to 60 days at 25°C. The mycelia from the margins of fungal colonies growing from the leaf fragments were transferred to fresh PDA and further purified by transferring hyphal plugs to new PDA plates. Thirteen endophytic fungi were selected from leaves of* C. spiralis*. These microorganisms were previously tested with phytopathogen controls (data not shown) and are preserved in Culture Collection from the Laboratory of General and Applied Microbiology at the Federal University of Tocantins, Brazil.

### 2.2. Molecular Identification

#### 2.2.1. Fungal DNA Extraction

Endophytic fungi were cultured on Sabouraud dextrose agar slants for 7 days at 25°C. The DNA extraction was performed according to the method described by de Hoog et al. [34]. Briefly, approximately 0.1 g of fungal mycelia was transferred to 1.5 mL tubes containing 400 *μ*L of lysis buffer (50 mM Tris-HCl, pH 8.0, 5 mM EDTA pH 8.0, 100 mM NaCl, 1% SDS) and kept at −20°C for 10 min. The fungi cells were mixed with 1 mm glass beads and agitated using a Vortex for 30 sec followed by a 30 sec interval on ice, then repeated. Cells were incubated for 30 min at 60°C with 5 *μ*L 20 mg/mL Proteinase K. The samples were then incubated for 10 min at 65°C with 162 *μ*L of CTAB solution (200 mM Tris-HCl, pH 7.5, 200 mM Na-EDTA, 8.2% NaCl, 2% CTAB). Following incubation, 570 *μ*L chloroform: isoamylic alcohol (24 : 1 v/v) was added, and the tubes were kept for 10 min on ice. The homogenate was centrifuged at 14,500 ×g for 10 min at 4°C. Then, the supernatant was transferred to a new tube, and 10% of the residual volume of 3 M sodium acetate was added. The mixture was then incubated on ice for 30 min. Precipitates were removed by centrifugation, and the DNA was recovered by isopropanol precipitation, washed with 70% (v/v) ethanol, allowed to air dry, and resuspended in 50 *μ*L of TE buffer (10 mM Tris-HCl, pH 8.0, and 0.1 mM EDTA). The concentration was measured by monitoring the UV absorbance at 260 nm using Nanodrop 2000 (Thermo USA), and the DNA was diluted to 10 ng/*μ*L with ultrapure water.

#### 2.2.2. DNA Amplification and Sequencing

DNA sequencing was performed using a BigDye Direct Cycle Sequencing Kit (Life Technologies). For PCR amplification, 1 *μ*L of diluted DNA (10 ng/*μ*L) was mixed with 5.0 *μ*L of BigDye Direct PCR Master Mix, 0.75 *μ*L of each forward ITS1-M13 (5′-TGTAAAACGACGGCCAGTTCCGTAGGTGAACCTGCGG-3′) and reverse ITS4-M13 (5′-CAGGAAACAGCTATGACCTCCTCCGCTTATTGATATGC-3′) primers at a 10 *μ*M concentration, which include the M13 universal primer sequences, and 2.4 *μ*L of ultrapure water. PCR cycling conditions were as follows: initial denaturation of 10 min at 94°C, followed by 35 cycles (30 s) of denaturation at 96°C, annealing for 15 sec at 62°C, and extension for 30 sec at 72°C. Following PCR, 3 *μ*L of BigDye Direct Sequencing Master Mix and 1 *μ*L of forward BigDye Direct M13 forward primer (provided) were added directly to the PCR sample, put back into the PCR thermal cycler, and incubated as follows: 15 min at 37°C, 2 min at 80°C, and 1 min at 96°C, followed by 25 cycles of 10 sec at 96°C, 5 sec at 50°C, and 75 sec at 60°C.

At the completion of the sequencing reaction, the sequencing products were purified following the ethanol/EDTA protocol for BigDye Terminator version 3.1 Cycle Sequencing Kit. Electrophoresis was performed on the Applied Biosystems 3500xL Genetic Analyzer. Electropherograms were proofread with the software BioEdit Sequence Alignment Editor (1997–2013). The sequencing results for each sample were analyzed using BLASTn (Basic Local Alignment Search Tool, version 2.215 of BLAST 2.0) to verify the results and to identify the fungus for each sample.

### 2.3. Crude Extract Acquisition

The 13 endophytes were multiplicated in PDA at 25°C ± 2 for 5 days. Subsequently, 6 mm discs were placed in the center of 4 Petri dishes with PDA agar and incubated at 25°C ± 2 for 14 days. These cultures were extracted (1 : 20 p/v) by hydroethanolic maceration (1 : 3 v/v) for 48 h at 25°C, under agitation. The samples were centrifuged at 2,400 ×g for 20 min at 8°C and then filtered. The solvent from each supernatant was removed on a rotary evaporator at 35°C, lyophilized, and stored in a desiccator without light. The same extraction process was performed for 4 sterile BDA Petri dishes that were used as negative control.

### 2.4. Antimicrobial Activity

#### 2.4.1. Microorganisms

All microorganisms were obtained from the American Type Culture Collection (ATCC, Rockville, MD, USA). Extracts were tested against the Gram-positive (G+ve) bacteria* Bacillus subtilis* 6623,* Enterococcus faecalis* 29212, and* Staphylococcus aureus* 29213; Gram-negative (G−ve) bacteria* Escherichia coli* 25922,* Pseudomonas aeruginosa* 27853,* Klebsiella pneumoniae* 700603, and* Salmonella enterica* subsp.* enterica serovar Typhi* 19430; and the yeasts* Candida albicans* 10231 and* C. parapsilosis* 22019.

#### 2.4.2. Antibacterial Susceptibility Test

The minimal inhibitory concentrations (MICs) of all extracts and the reference antibiotics tetracycline (Sigma, T3258) and penicillin (Sigma, P3032) were determined using microdilution techniques in Mueller-Hinton broth (Merck) following the protocol established for bacteria [35]. Inoculates were prepared in the same medium at a density adjusted to a 0.5 McFarland turbidity standard (10^8^ colony-forming units (CFU)/mL) and diluted 1 : 10 for the broth microdilution procedure. Microtiter plates were incubated at 37°C, and the MICs were recorded after 24 h of incubation. The lyophilized crude extracts of endophytes were diluted in dimethylsulfoxide (Sigma, D8418), filtered in sterile membranes, and tested at 1000, 500, 250, 125, 62.5, 31, 25, 7.8, 3.9, and 1.9 *μ*g/mL concentrations. The MIC was defined as the lowest concentration of crude extracts that inhibit the target microorganisms' growth.

The minimal bactericidal concentrations (MBCs) were determined from the results obtained in MIC. For this, an aliquot of 10 *μ*L from the wells that had inhibition was spread in Petri dishes with Mueller-Hinton agar (Fluka, 70191) and incubated at 37°C for 24 h. The MBCs were defined as the lowest concentration of crude extract that resulted in no growth when the treated culture was spread on antibiotic-free medium plates after the incubation. All tests were repeated and confirmed.

#### 2.4.3. Antifungal Susceptibility Test

The MICs of crude extracts were determined against yeasts using broth microdilution techniques, according to the method described by CLSI [36]. MICs were determined in RPMI 1640 medium (Sigma, R6504), pH 7.0. The starting inoculum was 1.0 × 10^6^ CFU/mL. Microtiter plates were incubated at 37°C in a dark humid chamber, and MICs were recorded after 48 h. The lyophilized hydroethanolic extracts from endophytes were diluted in dimethylsulfoxide (Sigma, D8418), filtered in sterile membranes, and tested at 1000, 500, 250, 125, 62.5, 31.25, 7.8, 3.9, and 1.9 *μ*g/mL concentrations. The MIC was defined as the lowest concentration of compounds without microorganism growth. Nystatin (Sigma, N6261) was used as the drug control. The minimal fungicidal concentrations (MFCs) were obtained from the MIC results. For this, an aliquot of 10 *μ*L from the wells that showed inhibition was spread in Petri dishes with Sabouraud agar (Merck, 105438) and incubated at 37°C for 24 h. The MFCs were defined as the lowest concentration of crude extract that resulted in no growth when the treated culture was spread on plates with antibiotic-free medium after the incubation. All tests were repeated and confirmed.

### 2.5. Antioxidant Assay

#### 2.5.1. Thin Layer Chromatography (TLC)

The antioxidant activity from the hydroethanolic extracts of the 13 fungi strains was evaluated by thin layer chromatography (TLC). The extracts were dissolved in a hydroethanolic solution (3 : 1 v/v) to achieve a final concentration of 20 mg/mL. The solution was loaded onto a TLC plate (20 × 20 cm, Merck) and eluted with ethyl acetate, formic acid, acetic acid, and water (100 : 11 : 11 : 27 v/v). A methanolic solution of 2,2-difenil-1-picrilhidrazil (DPPH) (Aldrich, D9132) at 0.2% was used to visualize the products. Quercetin (Sigma, Q0125) and ascorbic acid (Sigma, A0278) were used as standards. Positive samples showed yellow bands on a purple background in chromatograms.

#### 2.5.2. Determination of Antioxidant Activities by DPPH Method

Antioxidant activity was determined in accordance with the published methods [37, 38]. Ascorbic acid was used as a standard (2–10 *μ*g/mL, Sigma, A0278). Fungi extracts and negative controls were diluted to 25, 50, 100, 150, and 200 *μ*g/mL. A methanolic solution of DPPH (0.1 mM, 2 mL) was added to 1 mL of each dilution. For each experiment, solutions with 1 mL sample and 2 mL ethanol were used to establish a baseline. Controls were performed with 1 mL of ethanol and 2 mL of methanolic solution of DPPH (0.1 mM). These solutions were homogenized and kept in the dark for 30 min at 25°C, and the absorbance was obtained at 517 nm with a spectrophotometer (Biochrom, Model Biowave II). All tests were conducted in triplicate.

Antioxidant activity of each extract concentration was determined using the following equation:

(1) $$\begin{array}{c} \text{AA}\%=100-\left\{\left[{\text{Abs}}_{\text{sample}}-{\text{Abs}}_{\text{blank}}\right]\times \frac{100}{{\text{Abs}}_{\text{contr}}}\right\}, \end{array}$$

where AA is the total antioxidant activity, Abs_sample_ is the absorbance of samples or standard, Abs_blank_ is the baseline absorbance, and Abs_contr_ is the absorbance of the control solution.

Linear regression curves were obtained from the AA% results and their respective results and predictive equations were expressed as CE_50_.

#### 2.5.3. Determining the Antioxidant Activity by Ferric Reducing Antioxidant Power (FRAP)

FRAP analyses were performed in accordance with the published protocols [39]. An acetate buffer solution (0.3 M, pH 3.6), a 10 mM solution of 2,4,6-tris (2piridil)-s-triazine (TPTZ) in 40 mM HCl, and an aqueous solution of ferric chloride (20 mM) were prepared. The FRAP reagent was prepared few minutes before mixing the solutions for analysis (10 : 1 : 1 v/v).

The total antioxidant activity (TAA) for each of the crude extracts was determined from three methanolic dilutions (0.5, 0.25, and 0.125 mg/mL) and 6 aqueous dilutions using ferrous sulfate (Sigma-Aldrich, 61230) as a standard in 500 and 2000 *μ*M concentrations. For each sample, 0.1 mL of each dilution was added to 2.9 mL of FRAP and kept in the dark at 37°C for 30 min. The absorbance of each solution was determined at 593 nm using a spectrophotometer (Biochrom, Model Biowave II) and FRAP as an internal reference. From these results, calibration curves were calculated for each sample. The result of TAA from each sample was expressed in millimole equivalents of iron per mg of lyophilized fungi extract (mM Fe^+2^/mg Ext).

### 2.6. Chemical Evaluation of the Extracts by TLC

The secondary metabolites from the 13 hydroethanolic extracts and from the negative control were evaluated by TLC using different mobile phases and visualizing agents in aluminum sheets (20 × 20 cm) preactivated, covered by silicagel GF_254_ (Merck). The samples were dissolved in hydroethanolic solution (1 : 3 v/v). The sheets were placed in a chromatography chamber, eluted along a 16 cm path in one direction, and observed by luminescence (UV365 nm). Using the previously described techniques and parameters for identification [40] and identical standards, we were able to evaluate the following classes of secondary metabolites: alkaloids, with quinine standard (Aldrich, 145904) and caffeine (Calbiochem, 205548); monoterpenoids, sesquiterpenoids, and diterpenoids using a thymol standard (Sigma, T0501); triterpenoids and steroids, with a stigmasterol standard (Sigma, S2424); phenolic compounds, with a tannic acid standard (Sigma-Aldrich, 403040), rutin (Sigma, R5143), and scopoletin (Sigma, S104); coumarin, using scopoletin standards (Sigma, S104), and coumarin (Sigma, C4261); anthraquinones, naphthoquinones, and anthocyanins, with 4-hydroxyanthraquinone and lapachol standards (Aldrich, 142905); saponins and polyketides through a saponin standard (Sigma, 47036); thymol (Sigma, T0501) and stigmasterol (Sigma, S2424); condensed proanthocyanidins and leucoanthocyanidins, with a catechin standard (Fluka, 43412).

### 2.7. Quantification of Total Phenolic Compounds

The quantification of phenols in hydroethanolic extracts was realized using the Folin-Ciocalteu method [41], using tannic acid (Sigma-Aldrich, 403040) as a standard. An aliquot (150 *μ*L) from each sample (1.0 mg/mL) was added to 250 *μ*L of Folin-Ciocalteu reagent. 500 *μ*L of sodium carbonate solution (7.5%, w/v) was then added to the mixture. The sample was diluted to 5.0 mL with water and left to stand in the dark for 30 min at 25°C. Each sample had its absorbance verified at 760 nm in spectrophotometer (Biochrom, Model Biowave II) using water to establish a baseline.

The calibration curve was calculated using tannic acid at 0.1–0.5 *μ*g/mL as a standard. The total phenol content was expressed in milligram equivalents of tannic acid per gram of extract (mg EAT/g) using linear equations. The assays were conducted in triplicate, and the experimental design was completely analyzed using the Tukey test (ANOVA) with the ASSISTAT 7.6 beta software.

Pearson correlation analysis was performed using the BioEstat 5.3 software, and graphics were prepared with OrigimPro 8.6. We used the following ranges of the Pearson coefficient (*r*) to represent an absent or very weak correlation (0.00 to 0.19), a weak correlation (0.20 to 0.39), a moderate correlation (0.40 to 0.59), a strong correlation (0.60 to 0.79), and a very strong correlation (0.80 to 1) [42].

## 3. Results and Discussions

### 3.1. Molecular Identification

Molecular identification was achieved by sequencing the* Internal Transcribed Spacer* (ITS) region. Sequences with 99% or more similarity were considered to be from the same species. Those with similarities between 93% and 98% were considered to be from the same genus. Sequences below 93% similarity were considered to be a previously unidentified strain [43]. Among the thirteen endophyte fungi analyzed, 11 were identified as being from the* Phomopsis/Diaporthe*,* Cochliobolus*, or* Sordariomycetes* genus (Table 1).

The unidentified fungi (4426 and 4400) may represent new species, and further studies are needed to determine their phylogenetic classification.

### 3.2. Antibacterial and Antifungal Activities

The endophytic fungi extracts were tested against G+ve and G−ve bacteria and yeasts. These microorganisms were selected according to their medical importance. The minimum inhibitory concentration (MIC) of extracts was determined using the microdilution method (Table 2) [44].

In the literature, there are no representative criteria for the MIC of endophytic fungi extracts. Therefore, we used the criteria cited in Table 3 for comparison [45].

The MIC values obtained for* C. albicans* and* C. parapsilosis* show moderate activities for all of the extracts tested. The* CEBP1*,* CEP4*,* CEDp11*, and* CEC12* had a minimum fungicidal concentration (MFC) equal to 125 *μ*g/mL for both yeasts, showing a fungicidal effect in the* Phomopsis* sp.,* D. phaseolorum*, and* Cochliobolus* sp. extracts. All other extracts showed no fungicidal activity.

For the antibacterial evaluation, the tested extracts were active against G+ve and G−ve bacteria. Eight extracts were able to inhibit the growth of* B. subtilis*, with MIC between 62.5 and 125 *μ*g/mL.* B. subtilis* was the microorganism most inhibited by the tested extracts, followed by* P. aeruginosa*, which was inhibited by five extracts, and* S. enterica* and* E. faecalis*, which were both inhibited by two extracts. The lower MICs (62.5 *μ*g/mL), which are considered to be good activities, were obtained from* CEC12* against* S. enterica* and from* CEP5* and* CEDp11* against* B. subtilis*, the last of which showed an MBC of 62.5 *μ*g/mL. The other antibacterial assays did not report MBC. Strains* CEDp11* and* CEC12* inhibited more pathogens (*C. albicans*,* C. parapsilosis*,* P. aeruginosa*,* S. enterica*,* B. subtilis*, and* E. faecalis*).

Antimicrobial activities from endophytic fungi of* C. spiralis* have not previously been reported in the literature. Our results thus demonstrate the biotechnological potential of these strains.

### 3.3. Chemical Evaluation of Extracts

Different classes of secondary metabolites were found by evaluating the 13 fungi extracts. We observed variations between the detected compounds and the fungi species (Table 4). Particularly noteworthy are the presence of triterpenes in five extracts and the presence of steroids and coumarins and the absence of alkaloids and condensable tannins in all analyzed extracts.

**Figure 1:**
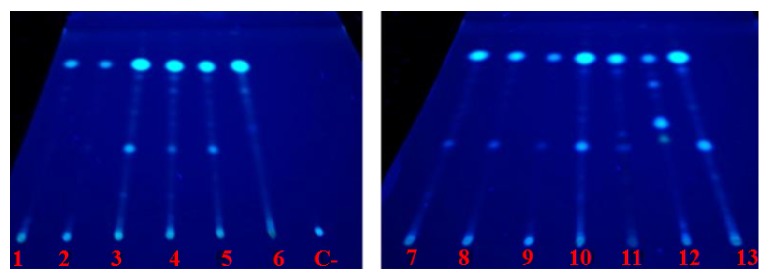
Fingerprints of 13 fungi extracts obtained by TLC observed by UV at 365 nm. Mobile phase: toluene : ethyl acetate (75 : 25 v/v). 1:* CEP1*, 2:* CEDp2*, 3:* CED3*, 4:* CED4*, 5:* CEP5*, 6:* CE6*, 7:* CED7*, 8:* CES8*, 9:* CE9*, 10:* CEP10*, 11:* CEDp11*, 12:* CEC12*, 13:* CES13*, C-: negative control.

We conducted TLC using different mobile phases for the 13 analyzed extracts. The best resolution among the compounds was observed with toluene : ethyl-acetate (75 : 25 v/v), observed by UV at 365 nm. The fingerprints of all fungi extracts showed compounds with common characteristics of coumarins by emission of blue or green fluorescence, and these results were confirmed by NEU visualization at UV365 nm (Figure 1).

The fingerprints show the presence of one complex matrix of compounds in the extracts. With respect to the molecular composition, we observed differences and a single profile for* CE6*,* CEDp11*,* CEC12*, and* CES13*, and we observed similarities between* CEP1* and* CEDp2*, the samples* CED3*,* CED4*, and* CEP5*, and the samples* CED7*,* CES8*, and* CE9*. It was also possible to observe variations in this matrix of compounds between different strains from* D. phaseolorum* species (*CEDp2 *and* CEDp11*).

Experiments that showed differences in the production of secondary metabolites were observed with* Streptomyces* sp. [46]. These results confirmed the importance of using fingerprint acquisition and chemical screening as a tool for the simple selection of chemical characteristics in bioprospection of fungi strains.

The coumarins are secondary metabolites found in different organisms such as vegetables, bacteria, fungi, lichens, and others. This class of compounds was related to several biological activities, including protease inhibition, acetylcholinesterase, K vitamin antagonism, antimicrobial, growth regulation, antiallergenic, antimalaric, antiviral, immunosuppression, hypolipidemic, hypotensor, antispasmodic, and antioxidant activities [30, 47–50].

To obtain more information about the chemical compounds observed in the fingerprints, we conducted the fractionation of the crude extracts by liquid-liquid partition, using dichloromethane as the nonpolar organic phase and water as the polar phase. Fractions were analyzed by TLC and compared with the crude extracts. The results from* CEDp11* and* CEC12* show the nonpolar nature of most of the compounds in the extracts, similar to the coumarins identified in previous assays.

**Figure 2:**
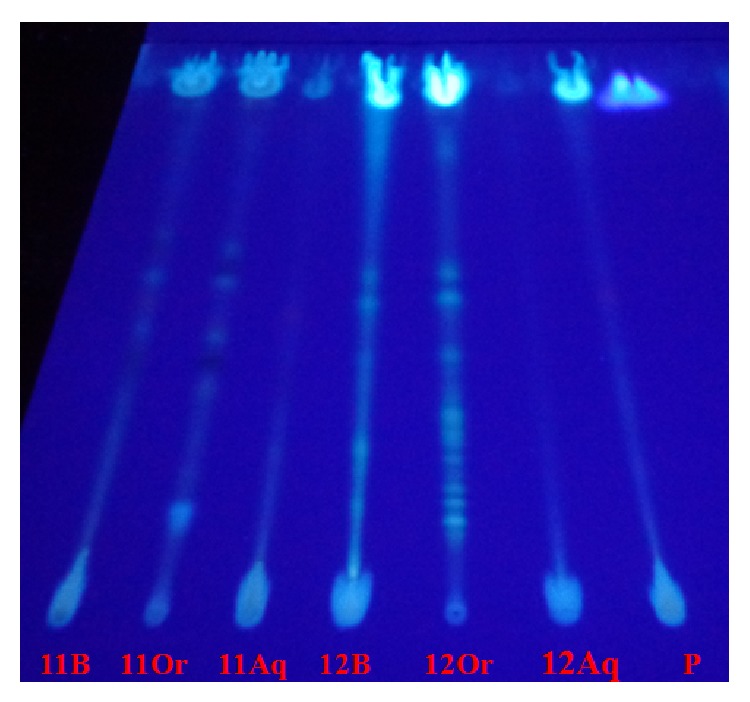
TLC of* CEDp11* and* CEC12* samples and their respective fractions, visualized at UV365 nm. Mobile phase: toluene : ethyl acetate (75 : 25 v/v). 11B: crude extract; 11Or: organic fraction; 11Aq: aqueous fraction; 12B: crude extract; 12Or: organic fraction; 12Aq: aqueous fraction; P: quercetin standard.

Further, the organic fractions show higher resolution among the compounds, being visualized in higher number when compared with the crude extracts. These data show the complex matrix of compounds present in the extracts obtained from these fungi species. This complex matrix can increase the interest in the possible biotechnological applications of these extracts and encourage the elucidation of the chemical structures of these molecules (Figure 2).

The* CEC12* fingerprint (ethyl acetate : toluene, 25 : 75 v/v) demonstrated 10 different fluorescent bands in the organic fraction. Bands 2, 3, and 6 (*R*_*f*_ 0.34, 0.42, and 0.62) represent compounds that are exclusive to this extract (Table 5).

When the mobile phase was used to separate anthracenic compounds, different from the other endophytic species, the sample* CEDp11* from* D. phaseolorum* showed peculiar compounds. The* CEDp11* fingerprint from the organic fraction had 5 unique compounds, 3 of which were colored by visible light in TLC (Table 5). The presence of orange and yellow bands (*R*_*f*_ 0.45, 0.55, and 0.91) is characteristic of anthraquinones. Typical fluorescence bands for coumarins were visualized in this sample (Table 5).

These results show the complexity of the analyzed samples, particularly the organic fractions of* CEDp11* and* CEC12*.

### 3.4. Quantification of Phenolic Compounds and Verification of Antioxidant Activity

The quantification of phenolic compounds from the 13 crude extracts and negative controls was conducted. Significant differences were found between the samples (*P* < 0.01) and the negative control (Table 6). Phenolic compounds have been very well known for their antioxidant properties, owing to their unique ability to act as free radical scavengers which, in turn, is an outstanding attribute of their unique biochemical structure [51].

TLC screening revealed the presence of antioxidant substances in all samples, by the presence of yellow bands in purple background resulting from the reduction of the DPPH radical. As a result of these analyses, the samples were submitted for characterization of their antioxidant activity using the DPPH and FRAP methods. The results were significant, particularly for* CEC12*,* CE6*,* CES13*,* CEDp11*, and* CEP1* (Table 6).

The average of values of antioxidant activities obtained from the FRAP and DPPH methods had a very strong Pearson correlation (*r* = 0.901, *P* < 0.05), showing that these methods have a positive correlation to the evaluated samples. This correlation suggests that these compounds act as hydrogen sources for DPPH radical and block the electron donation, resulting in iron complex reduction in the FRAP method. There was also a strong positive correlation between the phenolic compound contents in samples, according to the FRAP and DPPH results (*r* = 0.72, *P* < 0.05; *r* = 0.70, *P* < 0.05, resp.), possibly attributed to the antioxidant activity of phenolic substances, including coumarins.

Extracts that had higher antioxidant activities were submitted to liquid-liquid fractionation with dichloromethane. In the resulting organic fractions, the antioxidant activities increased (Table 6), showing the nonpolar characteristic of the antioxidant compounds present, as expected for coumarins. This is the first report of antioxidant activities from endophytic fungi from the Costaceae plants family. Coumarins that contain dihydroxyl groups in the ortho position, such as fraxetin (7,8-di-hydroxy-6-methoxycoumarin), esculetin (6,7-di-hydroxy-coumarin), and 4-methyl esculetin (6,7-di-hydroxy-4-methylcoumarin), are considered powerful lipid peroxidation inhibitors and can eliminate the superoxide anion radical to promote iron chelation. These proprieties make these substances very interesting as antioxidants, with possible applications in radial-free disease prevention [50].

The extracts from endophytic fungi from* C. spiralis* had antioxidant activities, and the important and complex chemicals can be explored for biotechnological purposes.

## 4. Conclusions

The biotechnological potential of pharmacological substances from endophytic fungi extracts of* C. spiralis* was demonstrated. All extracts had inhibitory activities against the yeasts* Candida albicans* and* C. parapsilosis*. Some extracts had antimicrobial activities against the following bacteria:* Bacillus subtilis*,* Pseudomonas aeruginosa*,* Salmonella enterica*, and* Enterococcus faecalis*. The antioxidant activities were measured by DPPH and FRAP, and all fungi had positive results.

The TLC results show very diverse chemicals in both the number of compounds and the metabolic class, whereas coumarins were present in all of the analyzed extracts. The fingerprint of CEDp11 (*D. phaseolorum*) was different from all others tested, including the sample CEDp2, which was from a different strain of the same fungi species, showing that each strain exhibits a unique chemical composition. These results have not been previously reported in endophytic fungi from the Costaceae plants present in Brazilian ecosystems.

## Figures and Tables

**Table 1:** Identification of endophytic filamentous fungi from *Costus spiralis *plant.

UFT code	Reference species	*GenBank* access number	% of identity	Number of bases	Species identification
4427	*Phomopsis *sp.	GU066693.1	99	564	*Phomopsis *sp.
4416	*Diaporthe phaseolorum *	JQ514150.1	99	603	*Diaporthe phaseolorum *
4432	*Diaporthe *sp.	FJ799941.1	98	602	*Diaporthe *sp.
4409	*Diaporthe *sp.	EF423554	99	534	*Diaporthe *sp.
4419	*Phomopsis *sp.	AY745986.1	99	561	*Phomopsis *sp.
4426	Not identified	—	—	—	Not identified
4417	*Diaporthe *sp.	FJ799941.1	99	602	*Diaporthe *sp.
4420	*Sordariomycetes *sp.	JX174146.1	99	549	*Sordariomycetes *sp.
4400	Not identified	—	—	—	Not identified
4435	*Phomopsis *sp.	EU977219.1	97	559	*Phomopsis *sp.
4405	*Diaporthe phaseolorum *	AY577815.1	99	602	*Diaporthe phaseolorum *
4410	*Cochliobolus *sp.	JQ753961.1	96	572	*Cochliobolus *ssp.
4403	*Sordariomycetes *sp.	JX174146.1	99	549	*Sordariomycetes *sp.

UFT code: Culture Collection of the Federal University of Tocantins, Brazil.

**Table 2:** Minimum inhibitory concentration (MIC, in *μ*g/mL) of crude extracts obtained from endophytic fungi isolated from *Costus spiralis* against yeasts and bacteria.

Extract code	Endophytic species	Yeasts		Bacteria						
		Ca	Cp	Ec	Pa	Kp	Se	Bs	Sa	Ef
*CEBP1 *	*Phomopsis *sp.	125	125	a	125	a	a	125	a	a
*CEDp2 *	*Diaporthe phaseolorum *	500	500	a	500	a	a	a	a	a
*CED3 *	*Diaporthe *sp.	500	500	a	a	a	a	a	a	a
*CED4 *	*Diaporthe *sp.	500	500	a	a	a	a	a	a	a
*CEP4 *	*Phomopsis *sp.	125	125	a	a	a	a	62.5	a	a
*CE6 *	Not identified	500	500	a	a	a	a	a	a	a
*CED7 *	*Diaporthe *sp.	250	250	a	a	a	a	125	a	a
*CES8 *	*Sordariomycetes *sp.	250	250	a	a	a	a	125	a	a
*CE9 *	Not identified	500	500	a	125	a	a	125	a	a
*CEP10 *	*Phomopsis *sp.	500	500	a	a	a	a	a	a	a
*CEDp11 *	*Diaporthe phaseolorum *	125	125	a	250	a	125	62.5	a	500
*CEC12 *	*Cochliobolus *ssp.	125	125	a	125	a	62.5	125	a	125
*CES12 *	*Sordariomycetes *sp.	250	250	a	a	a	a	125	a	a
*CECn *		a	a	a	a	a	a	a	a	a
Nystatin		1.9	1.9	—	—	—	—	—	—	—
Penicillin		—	—	—	—	—	1.9	3.9	3.9	3.9
Tetracycline		—	—	1.9	3.9	15.6	—	—	—	—

*Note.* Ca*: Candida albicans,* Cp*: C. parapsilosis, *Ec:* Escherichia coli, *Pa:* Pseudomonas aeruginosa, *Kp:* Klebsiella pneumoniae, *Se:* Salmonella enterica* subsp. *enterica serovar Typhi,* Bs*: Bacillus subtilis, *Sa:* Staphylococcus aureus*, and Ef:* Enterococcus faecalis.*

a: MIC/MFC/MBC above 1,000 *μ*g/mL; —: not available; *CECn: crude extract control negative.*

**Table 3:** Criteria of selection of positive antimicrobial activities of crude extracts.

MIC of crude extract	Result
Below 100 *μ*g/mL	Good antimicrobial activity
Between 100 and 500 *μ*g/mL	Moderate antimicrobial activity
Between 500 and 1000 *μ*g/mL	Weak antimicrobial activity
Above 1000 *μ*g/mL	Inactive

MIC = minimum inhibitory concentration.

**Table 4:** TLC results of secondary metabolites in the fungal extracts.

Crude extract code	Endophytic species	UV 365 nm^1^	NEU^2^	Chloridric vanillin^3^	KOH 10%^4^	KOH 10%^5^	Dragendorf^6^	Sulfuric vanillin^7^	Sulfuric vanillin^8^	Liebermann-Burchard^9^	Liebermann-Burchard^10^
*CEP1 *	*Phomopsis *sp.	(+)	(+)	(−)	(+)	(−)	(−)	(+)	(−)	(+)	(+)
*CEDp2 *	*Diaporthe phaseolorum *	(+)	(+)	(−)	(+)	(−)	(−)	(−)	(−)	(+)	(+)
*CED3 *	*Diaporthe *sp.	(+)	(+)	(−)	(+)	(−)	(−)	(+)	(−)	(+)	(+)
*CED4 *	*Diaporthe *sp.	(+)	(+)	(−)	(+)	(−)	(−)	(+)	(−)	(−)	(+)
*CEP4 *	*Phomopsis *sp.	(+)	(+)	(−)	(+)	(−)	(−)	(+)	(−)	(−)	(+)
*CE6 *	Not identified	(+)	(+)	(−)	(+)	(−)	(−)	(+)	(+)	(+)	(+)
*CED7 *	*Diaporthe *sp.	(+)	(+)	(−)	(+)	(−)	(−)	(+)	(−)	(−)	(+)
*CES8 *	*Sordariomycetes *sp.	(+)	(+)	(−)	(+)	(−)	(−)	(+)	(−)	(−)	(+)
*CE9 *	Not identified	(+)	(+)	(−)	(+)	(−)	(−)	(+)	(−)	(+)	(+)
*CEP10 *	*Phomopsis *sp.	(+)	(+)	(−)	(+)	(−)	(−)	(+)	(−)	(−)	(+)
*CEDp11 *	*Diaporthe phaseolorum *	(+)	(+)	(−)	(+)	(+)	(−)	(+)	(−)	(−)	(+)
*CEC12 *	*Cochliobolus *ssp.	(+)	(+)	(−)	(+)	(−)	(−)	(+)	(−)	(−)	(+)
*CES13 *	*Sordariomycetes *sp.	(+)	(+)	(−)	(+)	(−)	(−)	(+)	(−)	(−)	(+)
*CECn *		(−)	(−)	(−)	(−)	(−)	(−)	(−)	(−)	(−)	(−)

(+): positive; (−): negative; *CECn:* crude extract control negative.

^1^Coumarins; ^2^phenolic compounds; ^3^protoanthocyanins, leucoanthocyanins; ^4^coumarins (mobile phase to coumarins); ^5^anthraquinones, naphthoquinone, and anthocyanins (mobile phase to anthraquinones); ^6^alkaloids; ^7^phenols, sterols, terpenes, higher alcohols, polyketides, and saponins (mobile phase to saponins); ^8^phenols, sterols, terpenes, higher alcohols, polyketides, and saponins (mobile phase to mono-, sesqui-, and diterpenes); ^9^triterpenes (pink-reddish band); ^10^sterols (gray band).

**Table 5:** *R*_*f*_ values of bands from organic subfractions of *CEDp11 *(*D. phaseolorum*) and *CEC12* (*Cochliobolus* ssp.) in TLC.

Band	*R*_*f*_	Characteristic
*CEDp11* ^*^		
1	0.19	Blue fluorescence (UV 365 nm)
2	0.45	Orange (visible), red fluorescence (UV 365 nm)
3	0.52	Green fluorescence (UV 365 nm)
4	0.55	Orange (visible)
5	0.91	Yellow (visible), fluorescence (UV 365 nm)

*CEC12* ^**^		
2	0.34	Yellow fluorescence (UV 365 nm)—strong
3	0.42	Blue fluorescence (UV 365 nm)—strong
4	0.49	Blue fluorescence (UV 365 nm)—strong
5	0.55	Blue fluorescence (UV 365 nm)—strong
6	0.62	Blue fluorescence (UV 365 nm)—strong
7	0.68	Blue fluorescence (UV 365 nm)—weak
8	0.76	Blue fluorescence (UV 365 nm)—strong
9	0.92	Blue fluorescence (UV 365 nm)—weak
10	0.93	Blue fluorescence (UV 365 nm)—weak

^*^Mobile phase: ethyl acetate : methanol : water (100 : 13, 5 : 10 (v/v)).

^**^Mobile phase: ethyl acetate : toluene (25 : 75 (v/v)).

**Table 6:** Total phenol content and antioxidant activities by DPPH (CE_50_) and FRAP from hydroethanolic crude extracts and organic subfractions from endophytic fungi.

Code	Endophytic species	Average of total phenolic compounds concentrations (*μ*gTAE/mg)^*^	FRAP assay (*μ*M Fe_2_SO_4_/mg)^**^ crude extract	FRAP assay (*μ*M Fe_2_SO_4_/mg)^**^ organic subfraction	DPPH assay IC_50_ (*μ*g/mL)^**^ crude extract	DPPH assay IC_50_ (*μ*g/mL)^**^ organic subfraction
*CEP1 *	*Phomopsis *sp*. *	4.20 d	364.86 ± 2.38	481.6 ± 1.23	660.34 ± 2.71	456.76 ± 1.13
*CEDp2 *	*Diaporthe phaseolorum *	3.71 d	290.43 ± 1.21	NC	1136.91 ± 5.7	NC
*CED3 *	*Diaporthe *sp.	3.49 d	233.71 ± 0.23	NC	1375.96 ± 2.01	NC
*CED4 *	*Diaporthe *sp.	3.65 d	173.46 ± 0.34	NC	1309.00 ± 1.23	NC
*CEP4 *	*Phomopsis *sp.	3.58 d	120.05 ± 0.10	NC	1425.16 ± 2.10	NC
*CE6 *	Not identified	4.89 c	445.29 ± 0.98	547.14 ± 1.09	452.01 ± 0.14	213.56 ± 1.23
*CED7 *	*Diaporthe *sp.	3.68 d	120.25 ± 1.90	NC	1765.30 ± 0.76	NC
*CES8 *	*Sordariomycetes *sp.	3.65 d	143.89 ± 0.43	NC	1235.34 ± 2.10	NC
*CE9 *	Not identified	3.55 d	200.14 ± 0.87	NC	1014.75 ± 2.34	NC
*CEP10 *	*Phomopsis *sp.	3.71 d	133.74 ± 0.29	NC	1802.26 ± 1.23	NC
*CEDp11 *	*Diaporthe phaseolorum *	5.67 b	294.96 ± 1.09	437.2 ± 0.98	659.42 ± 0.76	423.86 ± 0.76
*CEC12 *	*Cochliobolus* ssp.	5.28 bc	471.15 ± 2.87	543.5 ± 0.58	378.01 ± 0.87	189.78 ± 1.23
*CES13 *	*Sordariomycetes *sp.	7.10 a	413.01 ± 1.76	523.6 ± 1.23	628.49 ± 1.56	399.98 ± 0.14
*CECn *		0.06 e	NA	NC	NA	NC
AA		—	—	—	6.41	6.41

CE_50_ = concentration able to reduce in 50% the DPPH radical; ^*^averages followed by the same letter are not significantly different (Tukey's test, *P* > 0.01); ^**^values average ± standard deviation, *n* = 3; NA = not active; NC: not conducted; *CECn* = crude extract control negative; AA = ascorbic acid; TAE = tannic acid equivalent.
